# A local mode picture for H atom reaction with vibrationally excited H_2_O: a full dimensional state-to-state quantum dynamics investigation

**DOI:** 10.1039/c5sc03472h

**Published:** 2015-09-29

**Authors:** Shu Liu, Dong H. Zhang

**Affiliations:** a State Key Laboratory of Molecular Reaction Dynamics , Dalian Institute of Chemical Physics , Chinese Academy of Sciences , Dalian 116023 , Liaoning , China . Email: zhangdh@dicp.ac.cn

## Abstract

A local mode picture was postulated some time ago to explain the negligible OD product excitation from H reacting with D_2_O in fundamental asymmetrical excitation: the D_2_O asymmetric stretch state is best thought as a linear combination of local mode stretches with the H atom reacting preferentially with the excited OD bond, but has never been verified. Here we report the first full-dimensional state-to-state study for the title reaction with H_2_O in the ground and the first symmetric and asymmetric stretching excited states. It is found that symmetric and asymmetric stretching excited states, which vibrate in very different ways in the normal mode picture, behave essentially identically in the reaction. More importantly, our calculations revealed that the reaction produces a small fraction of OH in the *v* = 1 state, with the population close to the relative reactivity between the ground and vibrationally excited states, and therefore it confirms the local mode picture for H_2_O in symmetric or asymmetric stretch states when reacting with an H atom.

## Abstract 2

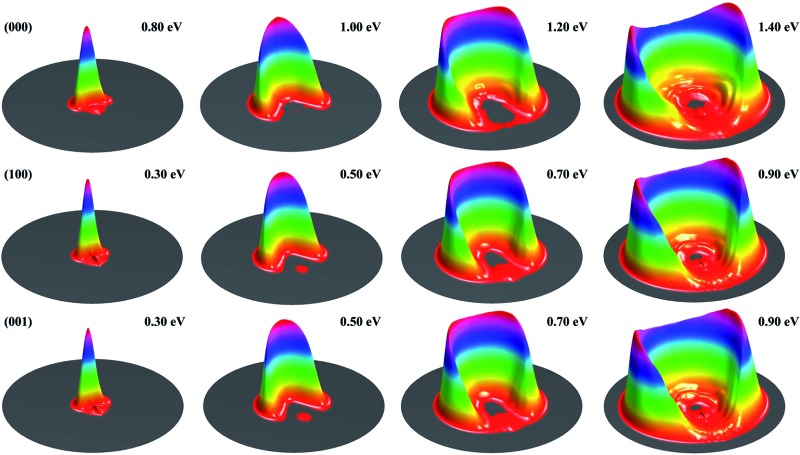
Here we report the first full-dimensional state-to-state study for the title reaction with H_2_O in the ground and the first symmetric and asymmetric stretching excited states..

## 

Molecular vibrations have profound effects on chemical reactivity. Consequently, it has been one of the central topics for chemical reaction dynamics to study how initial vibrational excitation influences reaction dynamics. Early studies on atom–diatom reactions led to the establishment of the well-known Polanyi rules, which state that vibrational energy is more efficient in promoting a late-barrier reaction than translational energy, whereas the reverse is true for an early barrier reaction.^1^ In past decades, substantial efforts have been devoted to understanding the dependence of reactivity on the initial excitation of a reactant mode for reactions involving polyatomic reactants.^2–9^ The H + H_2_O → H_2_ + OH reaction is the simplest system with different vibrational modes, and therefore has become a prototypical system for such a purpose. Crim, Zare, and co-workers carried out a series of experimental studies on the H + H_2_O/HOD/D_2_O reaction and its isotopically substituted analogies. Strong mode specific reactivity has been observed in the H + H_2_O/D_2_O reactions with H_2_O/D_2_O in overtone or fundamental vibrational excitations, and bond selectivity has also been demonstrated in the H + HOD reaction with a preferential cleavage of the HOD vibrationally excited bond.^10–15^ Theoretically, besides the quasiclassical trajectory (QCT) studies of Schatz *et al.*,^16–18^ there have been a number of quantum dynamics studies,^19–23^ which are in qualitative agreement with the experimental observations. Recently, Fu *et al.* calculated the exact coupled-channel (CC) integral cross sections (ICSs) for H_2_O and HOD initially in the first and second stretching excited states and simultaneous excitations of both bending and stretching modes on the YZCL2 PES, and observed strong mode specific and bond selected reactivity as discovered in the experiments.^24,25^

Another important question of mode specificity is how the initial vibrational excitation influences the product state distribution. The Crim group observed that the reaction of water molecules excited to the |04^–^ overtone state predominantly produces OH (*v* = 0), while the reaction from the |13^–^ state produces mostly OH (*v* = 1).^12^ The Zare group showed that for the D_2_O antisymmetric stretch fundamental states (001) and (011), the OD product is produced with little vibrational excitation [OD (*v* = 1) : OD (*v* = 0) ≈ 1 : 20].^15^ At first glance, such an OD (OH) product vibration distribution might seem surprising. In a normal mode picture, both OD bonds in D_2_O are equally excited in the symmetrically or antisymmetrically excited state. Breaking one OD bond by the incoming H atom should leave the other OD in a vibrationally excited state. Zare and coworkers postulated a local mode picture in which the D_2_O asymmetric stretch state is thought of as a linear combination of local mode stretches with the H atom reacting preferentially with the excited OD bond, leaving the other OD group in its vibrational ground state. However, this interesting local mode postulation has never been verified by theoretical studies.

In this letter, we report the first full-dimensional quantum differential cross-sections (DCSs) for the title reaction with initial nonrotating H_2_O in the (000), (100) and (001) vibrational states to investigate the initial vibrational excitation influences on the product state distribution and DCS. The H + H_2_O ↔ H_2_ + OH and its isotopically substituted reactions are the prototype for the theoretical study of tetra-atomic reactions, in much the same way that the H + H_2_ reaction served as the prototype for triatomics. Because three of the four atoms are hydrogens, the system is an ideal candidate for high quality *ab initio* calculations of a potential energy surface (PES), as well as for accurate quantum reactive scattering calculations. In the past few years, a number of PESs have been constructed with an increasing level of accuracy,^26–30^ making the system the first four-atom reaction with a quantitatively accurate PES. Through the development of the time-dependent wave-packet (TDWP) method, five-dimensional (5D) state-to-state DCSs were calculated for the H + H_2_O → H_2_ + OH reaction with the nonreactive OH bond length being fixed for the initial ground rovibrational state.^31,32^ Recently, the full-dimensional state-to-state DCSs were reported for the HD + OH → H_2_O + D and D_2_ + OH → HOD + D reactions with unprecedented agreement with experiments,^29,33,34^ declaring that theory is now able to compete with experimentation in studying simple four-atom reactions at the state-to-state level on given accurate PESs.

We employed the CXZ PES in our calculations,^30^ which is the most accurate and smooth PES available, constructed by the neural network (NN) method^35–37^ based on 17 000 *ab initio* points calculated at the UCCSD(T)-F12/AVTZ level of theory. The full-dimensional state-to-state quantum calculation is carried out by using the improved reactant–product-decoupling (RPD) scheme based TDWP method.^38,33^ The numerical parameters used in the reactant atom–triatom coordinates are as follows: the interaction region was defined by a rectangular box of [1.0,6.0]*a*_0_ in the *r*_1_ coordinate, and [1.0,6.0]*a*_0_ for the *R* coordinate. The number of vibrational basis functions used was 40 for the *r*_1_ coordinate. For the *R* coordinate, we used 40 sine discrete variable representation (DVR) points. The asymptotic region was defined from 6.0 to 12.0 *a*_0_ with 42 sine DVR points for the *R* coordinate and 8 vibrational basis functions for the *r*_1_ coordinates. The number of vibrational basis functions for the nonreactive OH is 12 for interactions and 7 for asymptotic regions. For the rotational motion, we used *j*_1max_ = 28 and *j*_2max_ = 50, and up to 9 K blocks to compute state-to-state reaction probabilities for *J* up to 40 to get converged DCSs. Continuous propagation in the product diatom–diatom coordinates only involves a total number of 120 sine functions for the translational coordinate *R*′ in a range of [3.0,15.0]*a*_0_, 5 vibrational basis functions for the H_2_ bond, and 3 vibrational basis functions for the OH bond. We used *j*_1max_ = 30 and *j*_2max_ = 18, and up to 11 K blocks for *J* = 40. To minimize the computational cost, the coordinate transformation is carried out at every 8 propagation time steps. Since one OH bond is treated as a nonreactive bond in this work, the reaction probabilities and cross sections should be doubled.

Fig. 1 shows the DCSs for the three initial states, each at four collision energies from the threshold energy, in terms of surface plots for the product translational energy and angle distributions for the reaction. It can be seen at the first glance that the first symmetric (100) and antisymmetric (001) stretching excited states have essentially identical DCSs. Just like the ground initial state, the DCSs for these two states are strongly backward peaked at the threshold energy. As the collision energy increases, the angular distribution becomes increasingly broader, and the peak position starts to shift gradually to a smaller angle, consistent with the fact that the title reaction is a direct reaction *via* an abstraction mechanism. At the same collision energy, the DCS for the ground state is very different from those for the vibrationally excited state. However, one may note that the DCSs at each column shown in Fig. 1 resemble each other very well, indicating that the angular distributions for these three states are similar at the same total energy (because the excitation energy for (100) and (001) states are about 0.454 and 0.466 eV, close to the collision energy difference between the ground and vibrationally excited state shown in the figure).

**Fig. 1:**
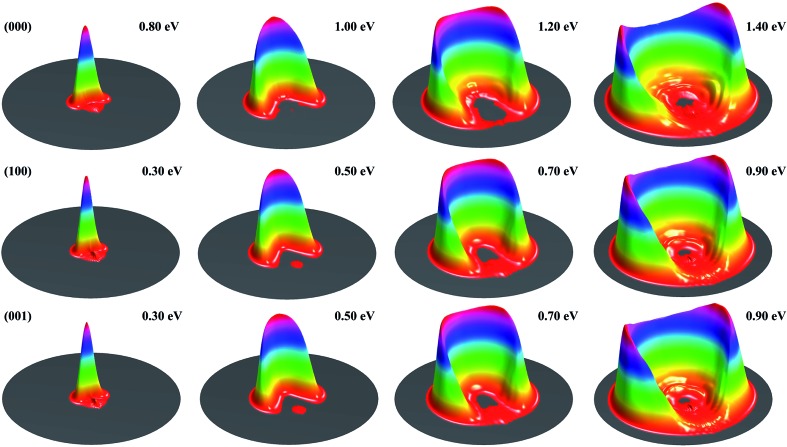
Surface plots for the product translational energy and angle distributions for the three initial states (000), (100) and (001) at several collision energies.

In order to examine the effect of the stretching excitation on the angular distribution in a quantitative manner, we present the total DCSs for the three states at collision energies of 0.8 eV, 1.0 eV, and 1.2 eV in Fig. 2(a). As seen, the vibrational excitations dramatically enhance the DCSs, but with the enhancement factor decreasing considerably as the collision energy increases. The behavior of angle distributions for the excited states is quite different from the ground state at the same collision energy, and the former are sideways peaked and have obvious forward scattering components at *E*_c_ = 1.2 eV. Moreover, the DCSs for the (100) state are only slightly larger than those for the (001) state at large scattering angles, indicating that one quantum in the symmetric or antisymmetric stretching excitation of H_2_O has nearly identical effects to DCS.

**Fig. 2:**
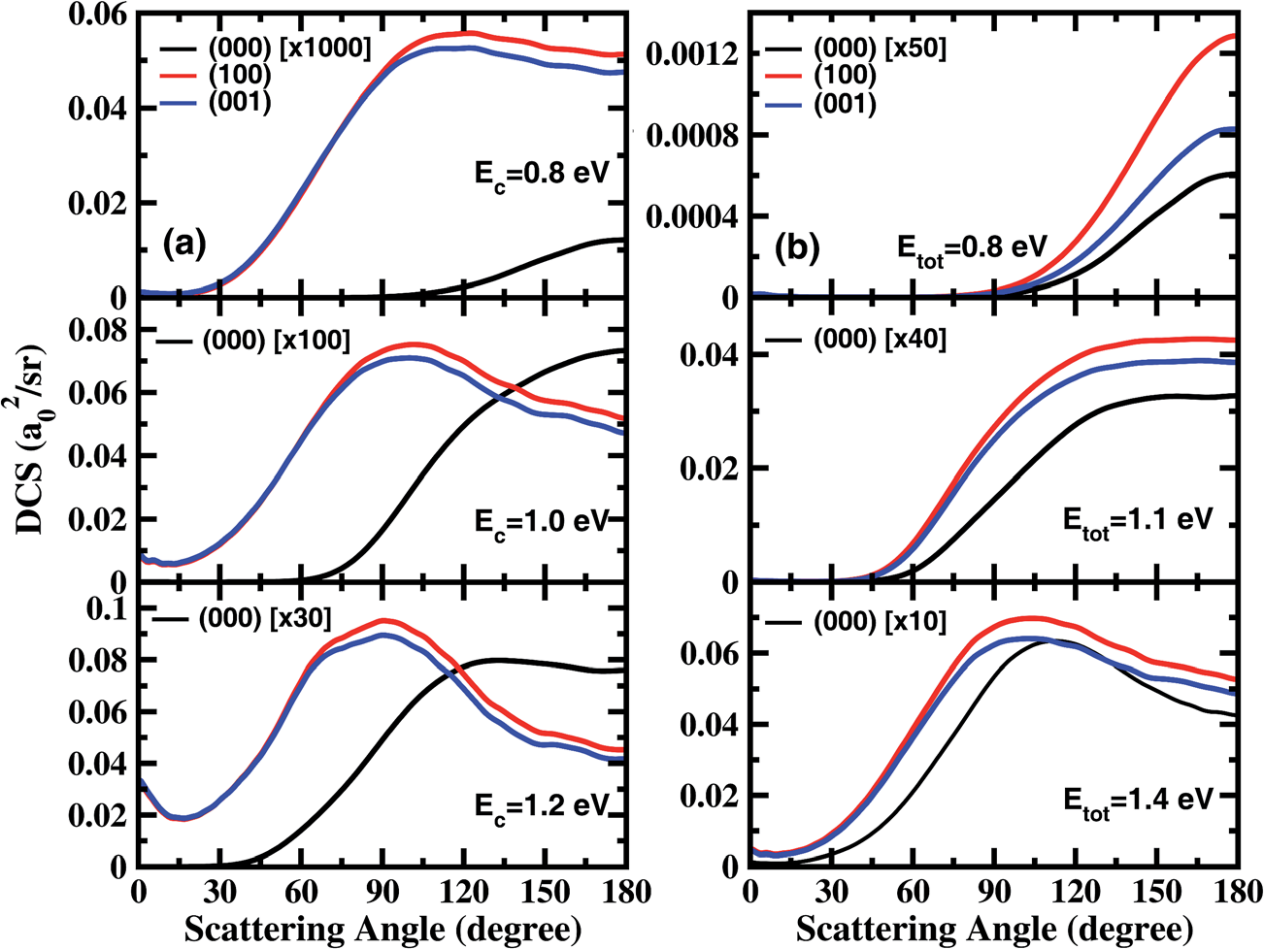
(a) Differential cross sections for the three initial states at collision energies of 0.8 eV, 1.0 eV and 1.2 eV; (b) differential cross sections for the three initial states at the total energies of 0.8 eV, 1.1 eV, and 1.4 eV.

Fig. 2(b) shows the total DCSs for the three states at total energies of 0.8 eV, 1.1 eV, and 1.4 eV, which are measured with respect to the energy of the H_2_O ground rovibrational state. Overall, the angle distributions for (000), (100) and (001) vibrational states are quite similar at the same total energy of reaction, although the excited states increase the cross section by at least 10 times relative to that for the ground state. As the total energy increases, the sideways scattering components increase substantially, and gradually become dominant. At *E*_tot_ = 1.4 eV, the angular distributions for the excited states are slightly broader with peaks at 103°, than that for the ground state with a peak at 113°. Therefore, the energy initially deposited in stretching vibrations is much more efficient than the translational energy at promoting the reaction, but has rather similar effects on product angular distribution as the translational energy.

Fig. 3 shows the product H_2_ (*v* = 1) and OH (*v* = 1) vibrational state populations for the three initial states as a function of total energy. It should be noted that the population for (000) state in Fig. 3(b) was multiplied by a factor of 5 before plotting. When H_2_O is in the vibrational ground state, both H_2_ and OH products are vibrationally very cold in the energy region considered here. There is a small fraction of H_2_ (*v* = 1) produced only for a collision energy higher than 1.25 eV with a population of 1.1% at 1.5 eV, and almost no OH (*v* = 1) produced. The initial vibrational excitations increase the branching ratios of H_2_ (*v* = 1) and OH (*v* = 1) channels at the same total energy. The population of H_2_ (*v* = 1) reaches 7.7% at *E*_tot_ = 1.65 eV. However, the OH vibration excited populations remain small for the initial (100) and (001) states.

**Fig. 3:**
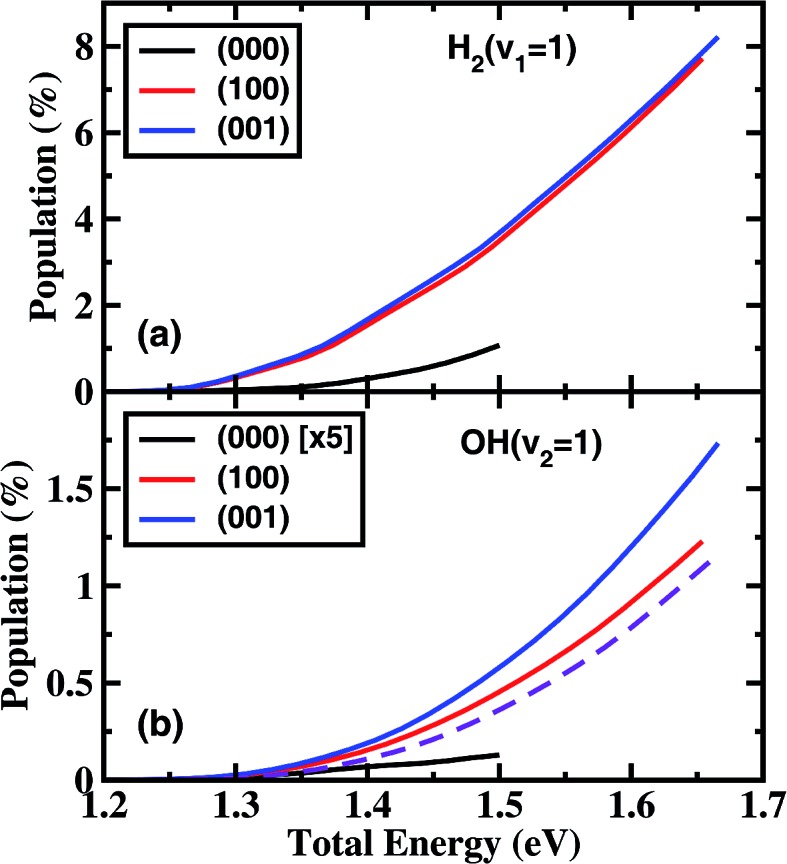
Populations of product H_2_ (*v* = 1) (a) and OH (*v* = 1) (b) vibrational states for the three initial states as a function of total energy, together with the relative reactivity between the OH (*v* = 0) and OH (*v* = 1) state (violet dashed line in (b)). Note the population of OH (*v* = 1) for (000) state shown in (b) was multiplied by a factor of 5 before plotting.

These small populations of OH (*v* = 1) clearly reveal that H_2_O fundamental stretching excitations produce vibrationally “cold” OH as the ground initial state, in agreement with the experimental observation of Zare and coworkers.^15^ In the normal mode picture, the (100) and (001) state correspond, respectively, to the symmetric and antisymmetric vibrational excitations of H_2_O with both OH bonds vibrationally excited. Breaking one OH bond by an incoming H atom will leave another OH bond vibrationally hot. However, in the local mode picture, the (100) and (001) states correspond to |10^±^ = 2^–1/2^(|10 ± |01), *i.e.* each OH bond is a superposition of the ground and first excited quantum states. When the incident H comes in, it will react with each quantum state independently without any effect from the non-reacting OH group if the non-reacting OH is a spectator, producing OH in the corresponding quantum state. Because the reactivity of the OH excited state is much higher than the ground state, the incident H atom tends to react with the excited state, producing OH mainly in the corresponding ground state. On the other hand, the H atom also reacts with the ground OH state, producing a small fraction of the OH (*v* = 1) state. The fraction of OH (*v* = 1) should be approximately equal to the relative reactivity between the OH (*v* = 0) and OH (*v* = 1) states. As can be seen from Fig. 3(b), the fraction of the OH (*v* = 1) population is very close to the relative reactivity. Therefore, the local mode picture works very well here, and the non-reacting OH bond does act as a spectator.

Fig. 4 shows the fraction of total available energy in the product channel going into the rotation and vibration excitation of H_2_ and OH as a function of total energy. For the ground state reaction, less than 25% of the available energy goes into the rotational motion of the products in the energy region considered here, and only a very small fraction appears as vibrational energy. The H_2_ rotation excitation carries away an increasingly large portion of the internal energy with the increase of collision energy, while the fraction of energy going into the OH rotation excitation essentially does not change with the collision energy. For the excited states reaction, the product rotational distributions are close to those for (000) at the same total energy, leading to a small difference in the fraction of energy. For the H_2_ vibration, the difference is bigger. The fraction obtained from vibrationally excited H_2_O is about a factor of 3.5 larger than that from the ground state H_2_O at *E*_tot_ = 1.5 eV.

**Fig. 4:**
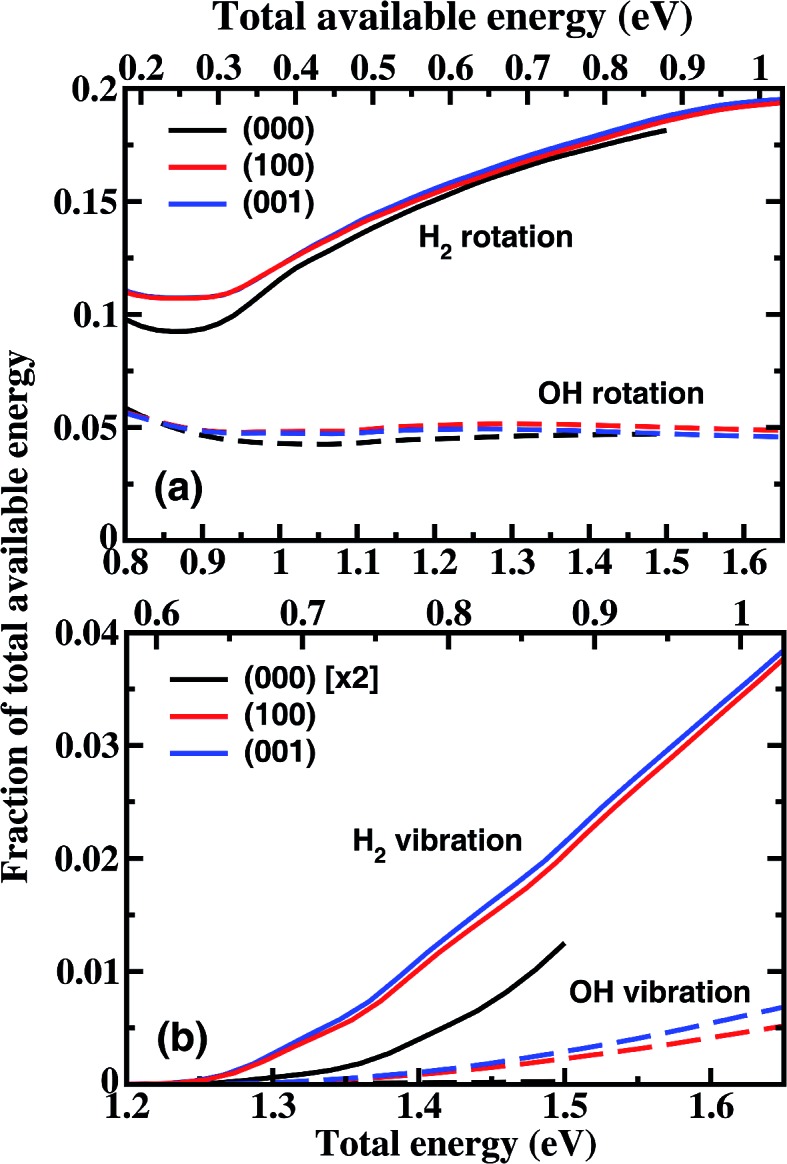
The fraction of the total available energy in the product channel going into the rotation (a) and vibration (b) excitation of H_2_ and OH as a function of the total energy. The corresponding total available energy in the product channel is also shown on the top of each panel. Note the fraction for OH vibration for the ground state was multiplied by a factor of 2 before plotting.

Therefore, our state-to-state quantum dynamics study revealed that the stretching excitation is much more efficient at promoting the reactivity than the translational energy, but has a rather similar effect on the angular distribution as the translational energy. The small populations of OH (*v* = 1) from (100) and (001) initial states can be explained clearly by the local mode picture of H_2_O vibration, and reveals that the non-reacting OH does act as a spectator in the reaction. More theoretical efforts should be put into studying higher levels of vibrational excitation and other X + H_2_O reactions in the future to further deepen our understanding of mode-specific chemistry.
